# Multiple-Locus Variable Number Tandem Repeat Analysis (MLVA) and Tandem Repeat Sequence Typing (TRST), helpful tools for subtyping *Staphylococcus lugdunensis*

**DOI:** 10.1038/s41598-018-30144-y

**Published:** 2018-08-03

**Authors:** Sandrine Dahyot, Jérémie Lebeurre, Xavier Argemi, Patrice François, Ludovic Lemée, Gilles Prévost, Martine Pestel-Caron

**Affiliations:** 1 Normandie Univ, UNIROUEN, GRAM EA2656, Rouen University Hospital, F-76000 Rouen, France; 2 Normandie Univ, UNIROUEN, GRAM EA2656, F-76000 Rouen, France; 3 Université de Strasbourg, CHRU de Strasbourg, VBP EA7290, Fédération de Médecine Translationnelle de Strasbourg (FMTS), Institut de Bactériologie, 3 rue Koeberlé, F-67000 Strasbourg, France; 4 Hôpitaux Universitaires, Maladies Infectieuses et Tropicales, F-67000 Strasbourg, France; 5 University of Geneva Hospitals, Genomic Research Laboratory, Service of Infectious Diseases, Geneva, Switzerland

## Abstract

*Staphylococcus lugdunensis* is an emergent virulent coagulase-negative *Staphylococcus* that is increasingly responsible for severe infections. In an attempt to generate informative sequence data for subtyping *S*. *lugdunensis*, we selected and sequenced seven polymorphic variable number of tandem repeats (VNTRs) to develop two new methods: a classic length-based multiple-locus VNTR analysis (MLVA) method and a tandem repeat sequence typing (TRST) method. We assessed their performances compared to two existing methods, multilocus sequence typing (MLST) and multivirulence-locus sequence typing (MVLST) for 128 isolates from diverse clinical settings and geographical origins. The clustering achieved by the four methods was highly congruent, with MLVA discriminating within clonal complexes as defined by MLST. Indeed, MLVA was highly discriminant compared to MLST and MVLST in terms of number of genotypes as well as diversity indexes. Sequencing of the seven VNTRs showed that they were stable, and analysis of sequence polymorphisms provided superior discriminatory power. The typeability, reproducibility, and epidemiological concordance of these new methods were excellent. Of note, no link between clustering and clinical settings was identified. This study demonstrates that MLVA and TRST provide valuable information for molecular epidemiological study of *S*. *lugdunensis*, and represent promising tools to distinguish between strains of homogenous lineages in this clonal species.

## Introduction

*Staphylococcus lugdunensis* is a coagulase-negative *Staphylococcus* (CoNS) belonging to the normal human skin microbiota^1,2^ that is increasingly recognized as a virulent pathogen in both community-acquired and healthcare associated infections^3,4^. This commensal bacterium has mainly been associated with serious infections, such as skin and soft tissue infections^5,6^, bone and joint infections^7,8^, but also bacteremia and endocarditis^9,10^. Thus, the pathogenicity of *S*. *lugdunensis* is closer to that of *S*. *aureus* than that of other CoNS; it may be related to virulence factors such as a fibrinogen binding protein^11^, a von Willebrand factor-binding protein^12^, synergistic hemolysins^13^ or a metalloprotease^14^.

Several molecular typing methods are currently available for *S*. *lugdunensis* strains such as macrorestriction analysis using pulsed-field gel electrophoresis^15^ and multilocus sequence typing (MLST)^16^. The latter is considered as the gold standard method for epidemiological typing and population genetic study of *S*. *lugdunensis*. Recently, we developed a multivirulence-locus sequence typing (MVLST) method, based on the sequence data of seven known or putative virulence-associated loci^17^. MVLST was significantly more discriminant than MLST, even allowing a trilocus sequence typing method for microepidemiological purposes. Phylogenetic analyses by MLST and MVLST revealed a clonal population structure, a mutational evolution of this pathogen, and a lack of hypervirulent lineages.

An interesting source of genetic polymorphism is provided by tandemly repeated sequences, known as variable number of tandem repeats (VNTRs), whose number of repetitions varies at different rates depending on the different loci and even alleles^18^. Molecular typing based on the analysis of repeat copy number at multiple VNTR loci, known as multiple-locus VNTR analysis (MLVA), is a genotyping method that is being used for strain comparison, and can also provide insights into population structure^19^. This method has already been successfully applied to many other bacterial species and showed a high level of discrimination^19–23^. Especially, measuring repeat polymorphisms in clonal microorganisms can provide a solid basis for genetic type assignment^24^. In addition, to increase the discriminatory power of MLVA, sequencing repeated sequences that display internal variability can be performed^25^.

The aims of this study were to identify VNTR loci and to sequence them in order (i) to develop the first length-based MLVA method and a sequence-based MLVA (tandem repeat sequence typing) method for *S*. *lugdunensis*, and (ii) to evaluate and compare the added value of these new methods with the existing methods MLST and MVLST in terms of *S*. *lugdunensis* strain discrimination within clonal groups.

## Results

### MLST and MVLST genotyping

To determine their diversity and whether they belong to major clonal complexes, we analyzed 128 *S*. *lugdunensis* clinical isolates by MLST and MVLST. Data for 20 isolates were derived from our previous studies^16,17^, while we performed MLST and MVLST analysis of 108 additional clinical isolates for the purposes of the present study.

**Figure 1:**
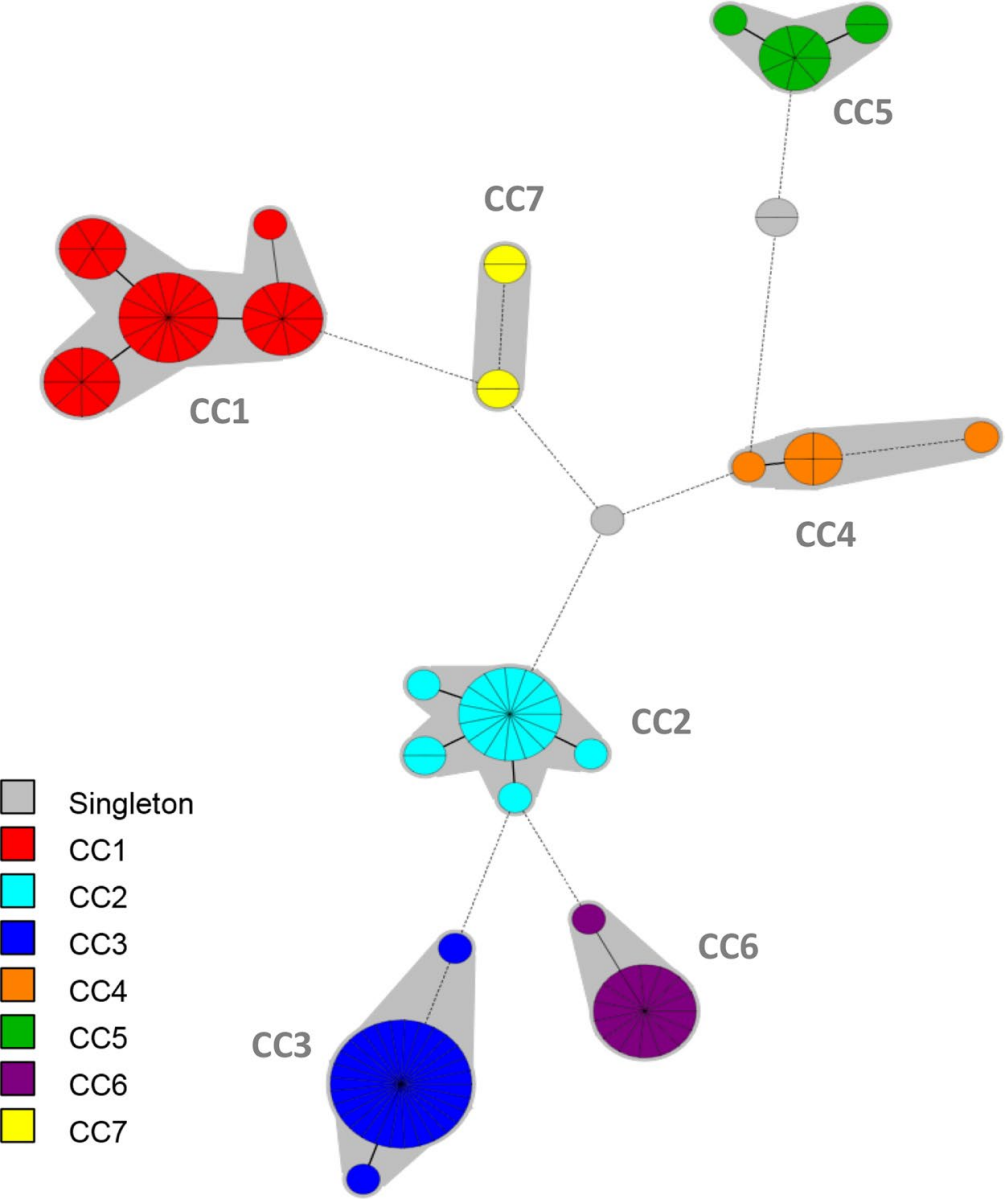
Minimum spanning tree of the 128 *S*. *lugdunensis* isolates typed by multilocus sequence typing (MLST). The MLST sequences were concatenated and analyzed in BioNumerics. Clustering of MLST profiles was done using a categorical coefficient. The colors used are based on clonal complexes (CCs). Each circle represents a sequence type and its size is proportional to the number of strains. Thick, short lines connecting two types denote types differing in a single locus; thin, longer lines connect double-locus variants; and dashed lines indicate the most likely connection between two types differing in more than two loci. Gray zones around circles delineate CCs. CCs are also indicated in characters e.g. CC1 denotes MLST complex 1.

MLST analysis on the 128 isolates identified 25 sequence types (STs). Two new STs, designated ST31 and ST32, were incorporated in the online database (http://bigsdb.web.pasteur.fr/staphlugdunensis). ST3 was the most common ST (n = 29), followed by ST2 (n = 15) and ST24 (n = 15). The 25 STs were distributed over 7 clonal complexes (CC1 to 7) and 2 singletons, as shown in the minimum spanning tree (Fig. 1). The main clonal complex was CC1 (n = 38), consisting of 5 STs (1, 6, 7, 12 and 15, with ST6 as the primary founder) followed by CC3 (n = 31) with 3 STs (3, 16 and 20 with ST3 as the primary founder).

MVLST performed on the 128 isolates revealed that the number of individual alleles for each of the three genes ranged from 10 (for SLUG_16930 and *atlL*_*R3*_) to 11 (for *isdJ*). Twenty-one trilocus virulence types (VT^T^s) were assigned; 3 major VT^T^s (VT^T^1, VT^T^15 and VT^T^16) were shared by 81 isolates. Five new VT^T^ were identified (VT^T^34 to 38). The clustering obtained by MVLST was similar to that observed by MLST. For instance, VT^T^15 and VT^T^16 corresponded to ST2 and ST3 respectively, VT^T^1 clustered into 4 STs (ST1, ST6, ST12 and ST15) from CC1 and 3 linked VT^T^s (VT^T^13, VT^T^34 and VT^T^37) belonged to ST24.

No correlation was found between genotype and clinical origin; for example, CC1 was shared by isolates from 8 different clinical contexts. Isolates recovered from hematogenic infections (blood or osteoarticular isolates) or from skin and soft tissue infections did not cluster in separate lineages. Nevertheless, an interesting trend was observed: 4 of 5 device-associated infection isolates belonged to CC3 (VT^T^16). Pathogenic and carriage isolates overlapped in their genotype distribution, with 4 of 7 CCs containing isolates from both sources. However, although carriage isolates did not cluster in a unique lineage, 59% (10/17) belonged to CC3 and 53% (9/17) belonged to VT^T^16.

### MLVA genotyping and clustering

Based on the *S*. *lugdunensis* genomes available in GenBank database, 7 VNTR loci were selected and evaluated on the validation panel of 30 isolates. Four VNTR loci were located in open reading frames; SLU2, SLU3 and SLU6 were located in genes encoding hypothetical proteins while SLU7 was located in a gene encoding an AraC family transcriptional regulator. Three VNTRs (SLU1, SLU4 and SLU5) were located in non-coding regions. The tandem repeat (TR) unit size of the 7 VNTRs was between 24 and 58 bp (Table 1), while the number of TRs ranged from 1 to 11. MLVA discriminated 19 MLVA types (MTs) among the 30 isolates. The 5 pairs of isolates considered as clonal by MLST were also found to be clonal by MLVA, attesting the epidemiological concordance of the method.

**Table 1:** Primers used for MLVA and TRST methods and characteristics of the 7 variable number of tandem repeat (VNTR) loci.

VNTR name	Primer sequences (5′−3′)	Annealing temperature	Genome coordinates^a^	Putative function	Repeat size (bp)	Copy no. range	MLVA method		TRST method		
							DI^b^	CI^c^ 95%	No. of allelles	DI^b^	CI^c^ 95%
SLU1	F: ATCTCAGGTAAGGATATTCGCATTG R: CACAATGTTTCGTATTGAATGGCTT	56 °C	2201528 2201881	Non-coding	57	1–3	0.531	0.500–0.562	6	0.656	0.609–0.702
SLU2	F: TTTATCCCACAATCATTCCTTGC R: GTTCCAGTCTCTTGTCAATTAGTTT	55 °C	2247710 2248186	Hypothetical protein	58	1–4	0.595	0.523–0.667	21	0.801	0.745–0.856
SLU3	F: CACAAGACAATAGTAACCAGAAAG R: CTTTATTATTTGGTTGATTCGTTGG	55 °C	1080477 1080983	Hypothetical protein	48	3–11	0.679	0.615–0.743	27	0.889	0.859–0.918
SLU4	F: ATATTTCGTATTGTTGGCTCG R: GAAGCGCATAGTGTAGATGA	53 °C	569800 569359	Non-coding	57	1–6	0.666	0.617–0.715	14	0.757	0.706–0.808
SLU5	F: TTTAACATCATTGCAGGTCG R: CAGCAGAATACCATTTCAATTACA	53 °C	2497844 2497405	Non-coding	57	2–5	0.479	0.393–0.566	19	0.849	0.819–0.879
SLU6	F: TACATTAAAGCTAGTTTGCAG R: AAATAAGTGAAGGACGTGATG	60 °C	2115216 2115837	Hypothetical protein	58	1–5	0.566	0.503–0.629	18	0.850	0.819–0.880
SLU7	F: CAATGATGATGCCTAACACCG R: AATTATGGATTATATCGAGCG	58 °C	2254827 2255435	AraC family transcriptional regulator	24	2–3	0.090	0.022–0.158	4	0.120	0.043–0.197

^a^Nucleotide coordinates in the reference strains *S*. *lugdunensis* N920143 chosen for primer design. ^b^DI: Simpson’s diversity index. ^c^CI: confidence interval.

The MLVA assay was then applied to the analysis of an expanded panel of 98 *S*. *lugdunensis* clinical isolates from highly diverse clinical situations. The 7 VNTR loci were amplified and sequenced for all 98 isolates. Diversity in the number of TRs varied among the various VNTR loci. The largest observed variation in VNTR size was found for SLU3 and spanned from 3 to 11 repeats (Table 1). MLVA discriminated 42 MTs among the 98 isolates. Four MTs were represented by more than 5 isolates: MT1 (n = 23), MT2 (n = 12), MT3 (n = 7) and MT4 (n = 6). Twenty-nine MTs were represented by only 1 isolate.

Finally, the 7 selected VNTRs enabled identification of 55 different MTs for the overall panel of 128 isolates tested in this study. A complete overview of these allelic profiles is presented in Table S4. Simpson’s diversity index (DI) was calculated for each of the 7 loci to evaluate their discriminatory power. The SLU3 locus showed the highest DI value (0.679), with the highest number of TR; the lowest DI value was observed for the SLU7 locus (0.090) (Table 1). MLVA combined 2 markers (SLU7 and SLU5) with a low discriminatory power (DI < 0.5) with 5 highly discriminant markers.

**Figure 2:**
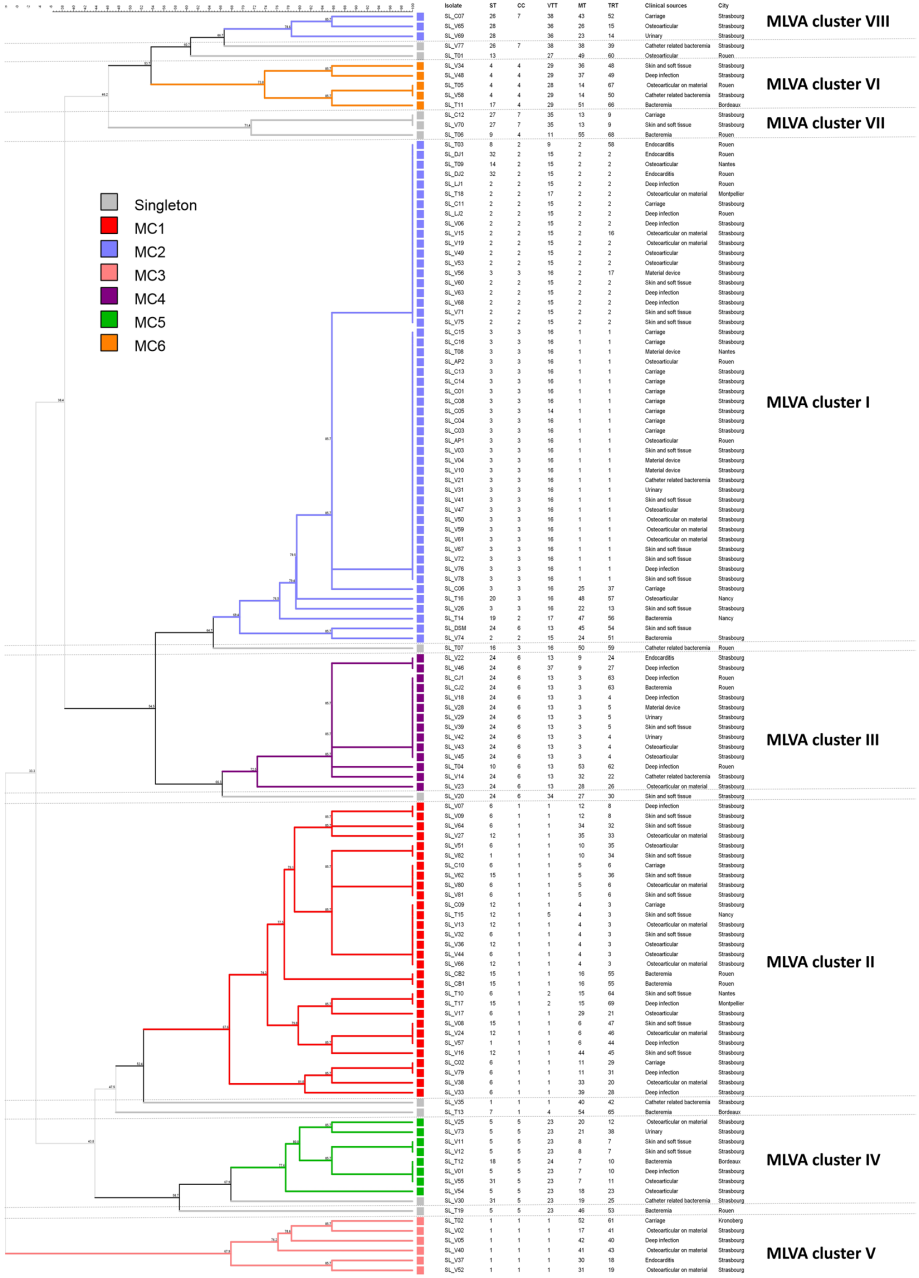
Multiple-locus VNTR analysis (MLVA) clustering of the 128 isolates of *S*. *lugdunensis* by the unweighted pair group method with arithmetic mean (UPGMA) method. The names of isolates, MLST sequence type (ST), MLST clonal complex (CC), MVLST trilocus virulence type (VT^T^), MLVA type (MT), TRST type (TRT), the clinical sources and the city of isolates are shown on the right. A cutoff value of 67% similarity was applied to define MLVA clusters (named MLVA cluster I to VIII). The colors used are based on MLVA complexes (MCs).

**Figure 3:**
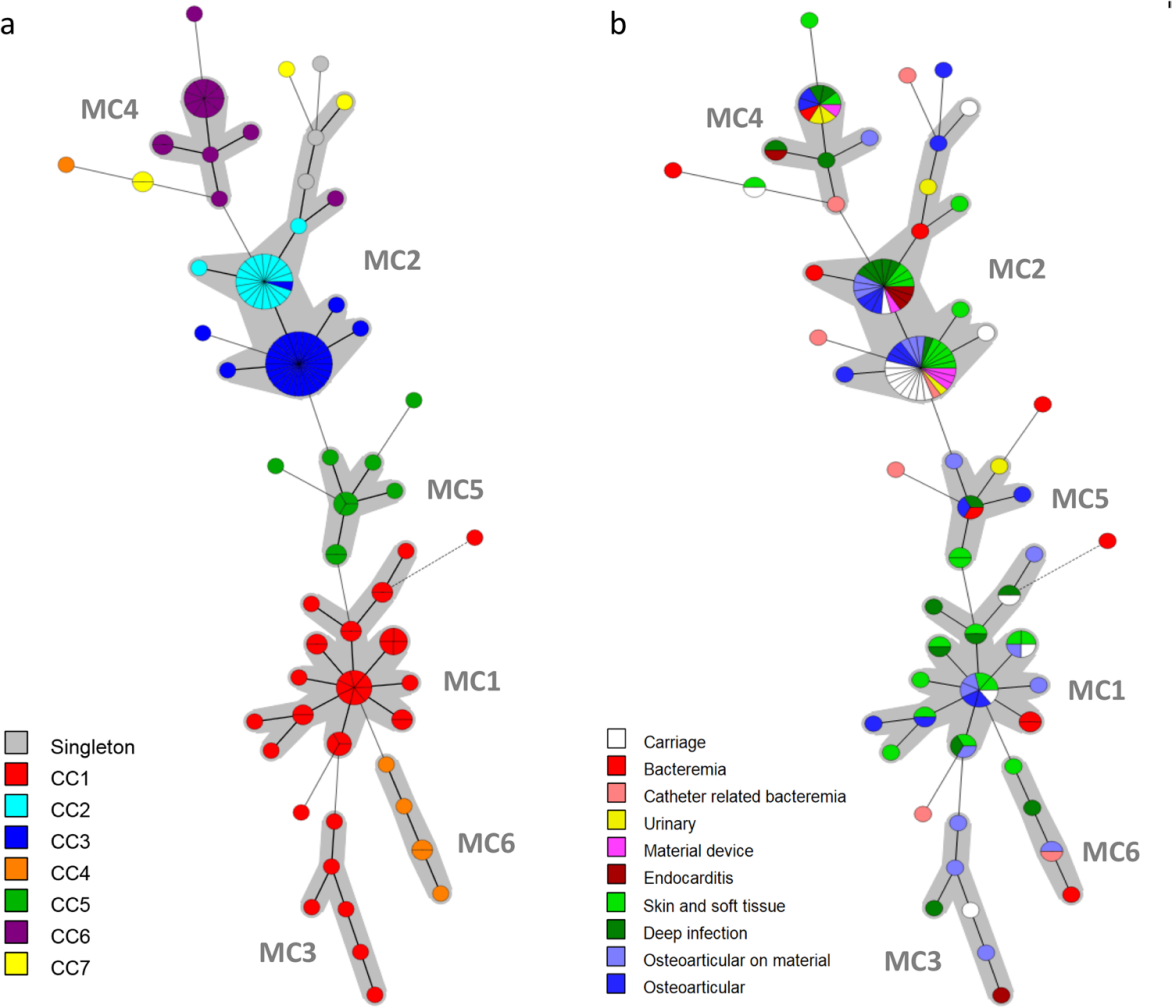
Minimum spanning tree of the 128 *S*. *lugdunensis* isolates typed by multiple-locus VNTR analysis (MLVA). The MLVA data were analyzed in BioNumerics. Clustering of MLVA profiles was done using a categorical coefficient. The colors used are based **(a)** on clonal complexes (CCs) defined by MLST and **(b)** on clinical contexts. Each circle represents an MLVA type. Thick, short lines connecting two types denote types differing in a single locus; thin, longer lines connect double-locus variants; and dashed lines indicate the most likely connection between two types differing in more than two loci. Gray zones around circles delineate MLVA complexes (MCs). MCs are also indicated in characters e.g. MC1 denotes MLVA complex 1.

The genetic relationships of the 128 isolates were deduced by the construction of an unweighted pair group method with arithmetic mean (UPGMA) tree (Fig. 2) and a minimum spanning tree (Fig. 3). For the minimum spanning tree analysis, MLVA complexes (MCs) were created if 6 of the 7 VNTR loci were identical. This analysis defined 6 different MCs (MC1 to 6) and 10 singletons (Fig. 3a). Two of the MCs, MC1 (30 isolates) and MC2 (54 isolates), were predominant. Of the 128 isolates analyzed in the study, 91% were part of an MC. With the UPGMA algorithm, an MLVA cluster was defined by a cutoff value of 67% similarity (Fig. 2). Eight MLVA clusters (cluster I to VIII), each containing more than 2 isolates, and 7 singletons were identified. The predominant cluster, cluster I, included 51 isolates. Overall, clusters were highly congruent with the 2 algorithms, UPGMA and minimum spanning tree.

The distribution of the isolates did not allow us to identify any correlation between MCs and clinical settings. However, 12 of 17 (70%) carriage isolates and 4 of the 5 device-associated infection isolates clustered into MC2 (Fig. 3b), as observed with MLST and MVLST.

Compared to MLST, MLVA resulted in a higher differentiation of the *S*. *lugdunensis* isolates as 55 MTs were clustered into 6 MCs as opposed to 25 STs into 7 CCs. Interestingly, MLVA efficiently subdivided some CCs such as CC1 and CC5 (Table 2). On the contrary, isolates of CC2, represented by 5 STs, were less discriminated by MLVA with only 3 MTs. Despite obvious differences, there was considerable congruence between the clustering results obtained by the 2 typing methods, as shown by the MLVA-based minimum spanning tree (Fig. 3a). Especially, the CC1 isolates clustered into 2 linked MCs (MC1 and MC3), and the CC4 and CC5 isolates clustered into MC6 and MC5, respectively.

**Table 2:** Abilities of the four typing methods to discriminate isolates within major *S*. *lugdunensis* clonal complexes (CCs) defined by MLST. ^a^DI: Simpson’s diversity index.

CC (no. of isolates)	MLST		MVLST		MLVA		TRST	
	No. of STs	DI^a^	No. of VT^T^s	DI^a^	No. of MTs	DI^a^	No. of TRTs	DI^a^
1 (37)	5	0.737	4	0.206	22	0.949	28	0.962
2 (18)	5	0.405	3	0.307	3	0.216	5	0.405
3 (30)	3	0.131	2	0.067	6	0.310	6	0.310
4 (6)	3	0.600	3	0.600	5	0.933	6	1.000
5 (10)	3	0.511	2	0.200	7	0.911	8	0.956
6 (15)	2	0.133	3	0.257	7	0.724	10	0.914
7 (4)	2	0.667	2	0.667	3	0.833	3	0.833
Total	25	0.898	21	0.845	55	0.933	69	0.943

### Tandem repeat sequence typing (TRST)

Sequencing of the alleles of the 7 VNTRs was performed to determine their internal variability and size homoplasy. Sequencing led to the identification of single nucleotide polymorphisms (SNPs) in the TRs of all loci, and showed that no insertion or deletion was present in flanking sequences. For each VNTR, alleles defined for the overall panel of 128 isolates ranged from 4 (SLU7) to 27 (SLU3) (Table 1). The Simpson’s DI calculated for the 7 VNTRs based on TRST ranged from 0.120 for SLU7 to 0.889 for SLU3 (Table 1).

Analysis of the 7 VNTR sequences revealed a total of 21 TRST types (TRTs) among the 30 panel test isolates; epidemiologically-related isolates belonged to the same TRT, which is in favor of an *in vivo* stability of the VNTR sequences. A total of 69 TRTs were identified for all the 128 isolates, suggesting that TRST is slightly more discriminant than MLVA (55 MTs). A complete overview of these allelic profiles is presented in Table S4. TRT1 was the most common (n = 26), followed by TRT2 (n = 16) and TRT3 (n = 7). Sixty-one TRTs were represented by only 1 isolate. A TRST cluster was defined by a cutoff value of 91% similarity with the UPGMA algorithm (Fig. S2). Nine TRST clusters (cluster I to IX), each containing more than 2 isolates were identified. The largest TRST cluster (cluster I) comprised 51 isolates represented by 11 TRTs. As illustrated by the UPGMA tree, these isolates belonged to the MC2 (Fig. S2).

**Figure 4:**
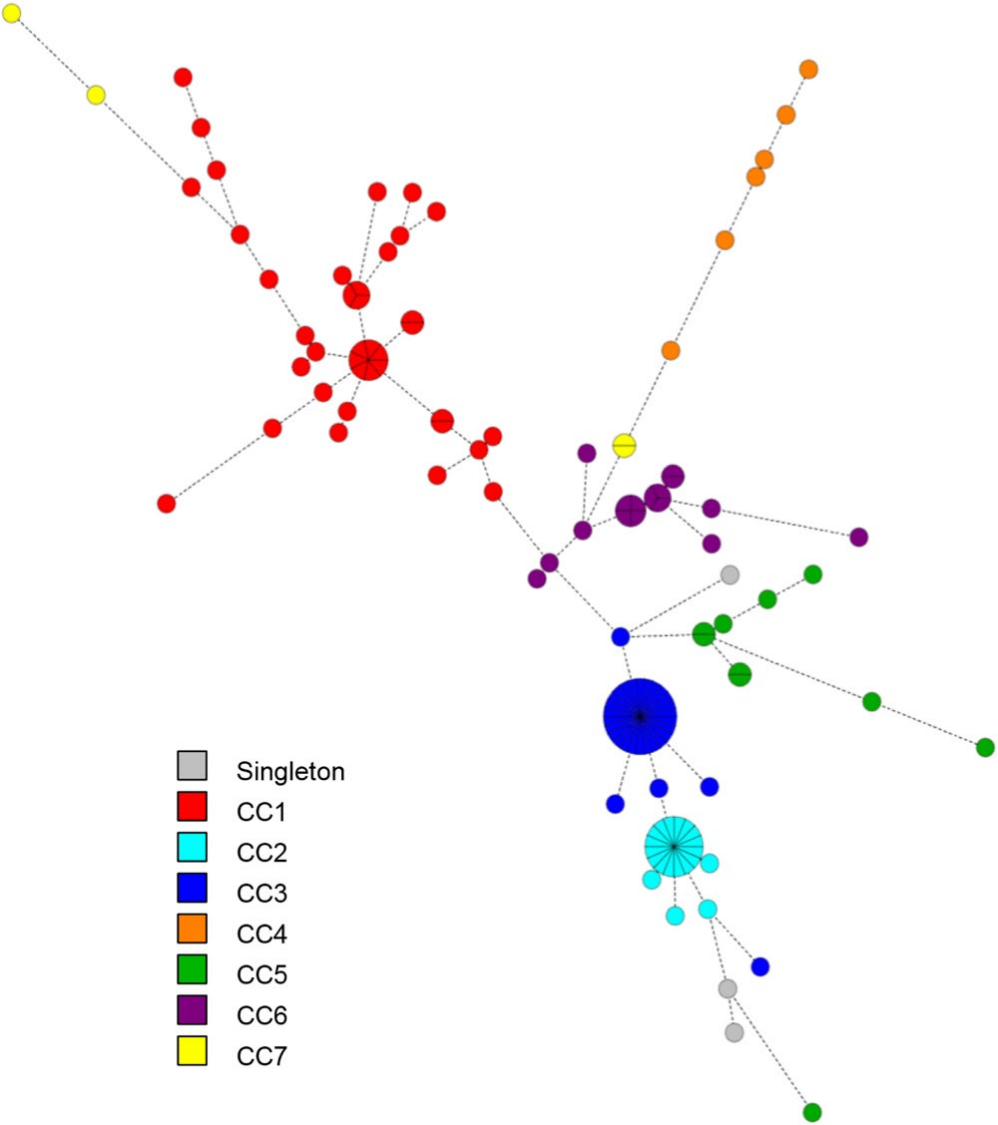
Minimum spanning tree of the 128 *S*. *lugdunensis* isolates typed by tandem repeat sequence typing (TRST). The VNTR sequences were combined into a composite dataset and analyzed in BioNumerics. Clustering of MLVA profiles was done using a categorical coefficient. Each circle represents a TRST type. The colors used are based on clonal complexes (CCs) defined by MLST. Thick, short lines connecting two types denote types differing in a single locus; thin, longer lines connect double-locus variants; and dashed lines indicate the most likely connection between two types differing in more than two loci.

Figure 4 illustrates the distribution of the distinct CCs onto the TRST diversity, similar to that obtained by MLST. Notwithstanding, as MLVA, TRST showed a variable level of discrimination according to CCs (Table 2). For example, TRST was highly discriminant for CC1 (28 TRTs *versus* 5 STs) and for CC6 (2 STs *versus* 10 TRTs).

As for MLVA, no correlation was observed between TRTs and clinical settings, but the majority of carriage (n = 11) and device-associated infection (n = 4) isolates remained clustered (TRST cluster I). Of note, 23 isolates collected from Strasbourg University Hospital between November 2013 and March 2016 could not be distinguished by both MLST (ST3) and MLVA/TRST (MT1/TRT1). As a fact, they could be considered as a clone. Interestingly, 14 isolates from 7 different clinical settings and 9 of the 16 carriage isolates belonged to this clone.

### Discriminatory power and concordance between typing methods

To assess the respective discriminatory power of the MLST, MVLST, MLVA and TRST methods, we excluded 5 of the 10 isolates that were isolated from the same patient and that were shown to be identical by all typing methods^26^. Simpson’s DI calculated on the basis of the 123 remaining unrelated isolates showed that MLVA (DI_MLVA_ = 0.933) was more discriminatory than MLST (DI_MLST_ = 0.898) and MVLST (DI_MVLST_ = 0.845) (Table 3). Moreover, discriminatory power contributed by the sequencing of the VNTR loci (DI_TRST_ = 0.943) was higher than the analysis of the size of the VNTRs.

**Table 3:** Simpson’s diversity index of the four typing methods. ^a^DI: Simpson’s diversity index. ^b^CI: confidence interval.

Typing method	Number of types	DI^a^	CI^b^ 95%
MLST	25	0.898	0.871–0.925
MVLST	21	0.845	0.809–0.880
MLVA	55	0.933	0.905–0.961
TRST	69	0.943	0.915–0.971

Table 2 shows Simpson’s DI of the different methods according to the CCs defined by MLST. The higher level of discrimination of MLVA and TRST was particularly observed within the CC1 isolates, with DI of 0.949 and 0.962 respectively, compared to MLST (DI = 0.737). Conversely, MVLST had a limited discriminatory power (DI = 0.206) for isolates of CC1.

The adjusted Wallace coefficient (AW) of MLVA, compared with that of MLST and MVLST, showed that MLVA had high congruence with MLST (AW = 0.826) and MVLST (AW = 0.815) (Table 4). This indicates that isolates from a given MT will have an 83% probability of sharing the same ST. Conversely, the congruency of MLST with MLVA indicates that isolates from a given ST will have only a 52% probability of belonging to the same MT. This confirmed that MLVA was more discriminatory than MLST. Moreover, the predictive ability of TRST for *S*. *lugdunensis* clustering was highly congruent with MLVA typing, and the probability that a pair of isolates with the same MT also shared identical TRT was 84% (Table 4).

**Table 4:** Adjusted Wallace coefficients and 95% confidence interval of the four typing methods.

	MLST	MVLST	MLVA	TRST
MLST		0.858 (0.756–0.959)	0.524 (0.407–0.641)	0.482 (0.368–0.597)
MVLST	0.531 (0.445–0.618)		0.320 (0.228–0.412)	0.289 (0.199–0.380)
MLVA	0.826 (0.736–0.916)	0.815 (0.677–0.953)		0.838 0.755–0.922)
TRST	0.906 (0.834–0.979)	0.878 (0.742–1.000)	1.000 (1.000–1.000)	

### Stability of VNTR loci

Examination of VNTR loci stability revealed that, for each isolate, identical MLVA profiles (in terms of TR number and sequences) were generated for the original isolates and their 10^th^, 20^th^ and 30^th^ subcultures, indicating that the 7 VNTRs were stable.

### Linkage disequilibrium

A quantitative analysis of the linkage between alleles from the 7 VNTR loci was performed by calculating the standardized index of association (*I*_A_^S^). The linkage disequilibrium was shown to be significant in the LIAN 3.7 analysis, indicating a clonal population structure for the *S*. *lugdunensis* isolates. *I*_A_^S^ was calculated at 0.1819 for MLVA and 0.4639 for TRST at a significance of *P* < 1.00 × 10^−5^.

## Discussion

Because *S*. *lugdunensis* is more virulent than other CoNS^27^, MLST and MVLST were developed to characterize the links between within-species genetic variations and characteristics, such as pathogenic potential, virulence, and epidemiology. To further delineate the lineages and clonal groups previously described by these typing methods, MLVA has attracted intense interest. In this study, we developed a classic seven loci MLVA typing method and a sequence-based MLVA method (TRST), and compared them with MLST as a reference method and with the virulence-associated trilocus method (MVLST). The relevance of these new molecular tools was investigated against a genetically diverse set of 128 *S*. *lugdunensis* isolates collected from various clinical settings and geographical origins.

One hundred TR sequences contained in the *S*. *lugdunensis* genomes available in GenBank database were initially identified *in silico*. Finally, seven VNTRs were selected because they met the preconditions described in the methods section and for their discriminatory power. PCR amplification and sequencing of the seven VNTRs were achieved for all the 128 isolates tested representing a typeability of 100%. MLVA proposes here a set of markers with different DIs (0.090 to 0.679). Markers with a moderate DI (<0.5), presumably reflecting their slow rate of evolution, define clusters. Conversely, VNTRs displaying more diversity and more rapid evolution reflect variability within clusters. Our MLVA method is similar to those proposed for *S*. *aureus*^20^ or *S*. *epidermidis*^21^, in terms of number of VNTRs and diversity indexes. Of note, 5 of the 7 VNTRs presented similarities (80 to 90% Blast identity) with other staphylococci, including *S*. *aureus* and *S*. *haemolyticus*, but the 7 primers are specific to the species *S*. *lugdunensis* excluding the possibility of false positive amplifications.

In the midst of all VNTRs identified with Tandem Repeats Finder software, some have been eliminated because fragments were too long to be amplified and sequenced in standard PCR conditions. For example, some were located in genes encoding putative surface-exposed proteins, like *sls* genes, as described by Heilbronner *et al*.^28^. However, these VNTRs could be attractive because the number of repeats is possibly more subjected to selective pressure. Indeed, previous MLVA studies of *Staphylococcus*^21,29^ and *Streptococcus agalactiae*^22^ found high variability in genes encoding Sdr adhesins and FbsA surface protein, respectively. Thus, these targets would be interesting to include in an MLVA method based on a whole genome sequencing approach.

We compared MLVA with the widely used MLST and the alternative trilocus MVLST. This latter method allowed us to define nearly the same number of profiles as MLST and was consistent with MLST, as unique STs or their single locus variants (SLVs) and double locus variants (DLVs) were found in each VT^T^ group. Thus, this comparison confirms the coevolution of virulence-associated loci studied with housekeeping genes as shown previously^17^, and validates the use of the trilocus method as a good epidemiological tool while significantly reducing costs.

Analysis of the seven VNTRs on the overall panel of isolates revealed that MLVA was highly discriminant compared to MLST in terms of number of profiles as well as discriminatory index. This could be explained by the fact that VNTRs evolve more rapidly than housekeeping genes, limiting the MLST resolution. However, although these two methods measure different evolutional mechanisms at multiple genomic loci, similar clustering was obtained, probably due to the rarity of recombination among *S*. *lugdunensis* isolates^16^. Thus, our findings suggest that the loci targeted by MLVA provide a robust measure of genetic relationships. The greater discriminatory index of MLVA made it possible to distinguish between isolates within CCs defined by MLST, but this appeared highly CC-dependent. Indeed, some clones, especially CC1, showed a significant diversity with many genotypes identified by MLVA and TRST. On the contrary, CC2 was less discriminated by these methods, suggesting that CC2 has emerged more recently than CC1. The higher discriminatory power of MLVA in some lineages defined as clonal by MLST has previously been described for several other species such as *Listeria monocytogenes*^23^ and *Streptococcus agalactiae*^22^.

On the other hand, MLVA is generally considered as an unreliable phylogenetic method because of the high frequency of the independent evolution of the same repeat array sizes in different isolates (*i*.*e*. size homoplasy)^30,31^. Thus, sequencing was selected not only as a method for repeat number verification, but also to detect single nucleotide polymorphisms in addition to allele size, SNPs being less prone to homoplasy^32^. It allowed us to propose a TRST method, which proved more discriminating than MLVA. Indeed, 55 MTs were identified by analyzing repeat array sizes, whereas 69 TRTs were identified by sequencing, indicating that a quarter of these alleles were misclassified by conventional MLVA. Considering that the growing use of whole genome sequencing, with its continuously decreasing costs and increased rapidity^33^, the present TRST method offers an additional tool for a worldwide traceable picture of *S*. *lugdunensis* clones. New alleles and TRST types will be certainly identified by *in silico*-TRST analysis as new genome sequences become available, contributing to a global evaluation of *S*. *lugdunensis* epidemiology.

MLVA and TRST have strong potential for inter-laboratory comparisons, providing accurate and reproducible data that can easily be exchanged. We have proved that the VNTRs studied were stable during laboratory subcultures, in terms of number of repeats as well as nucleotide sequences, and found complete reproducibility. Furthermore, the epidemiological concordance of the related strains has been validated. Thus, our new MLVA and TRST methods fulfill a number of criteria recognized as important for the successful implementation of a typing method and the interpretation of its results^26^. Here, the use of seven VNTRs resulted in high discriminatory capacity. Interestingly, the combination of only five markers (SLU1, SLU2, SLU3, SLU4 and SLU6) resulted in a highly similar clustering of isolates (44 MTs) while slightly decreasing the discriminatory capacity (DI = 0.920). This simpler version of the MLVA method could allow potential future use of multiplex PCR assays with fluorescent primers and determination of allele size by capillary electrophoresis.

In this study, no strong correlation was observed between genotypes and clinical settings of the 128 isolates. This could suggest that host susceptibility plays an important role in the propensity of isolates to cause disease, and/or that the virulence potential of isolates is not reflected by MLST or MLVA data. This has been described for *S*. *aureus*, for which MLST data do not provide any information regarding the pathogenicity of isolates, because changes in virulence correspond primarily to changes in the accessory, rather than core genome^34^. Like *S*. *aureus*, *S*. *lugdunensis* evolution is predominantly clonal^16,17^, therefore the study of the accessory genome could contribute to a better understanding of the genetic basis of these different clinical behaviors. On the other hand, the majority of carriage isolates and those collected from device-associated infections of this work were distributed in one complex whatever the typing method. Thus, it would be interesting to analyze a larger number of worldwide invasive and carriage *S*. *lugdunensis* isolates to measure the ratio of pathogenic to carrier strains for the various lineages. This ratio, combined with the study of the accessory genome, would help to investigate the existence of successful commensal clones.

As previously reported in other studies^4,16^, CC1 (n = 31) and CC3 (n = 26) were the most common clonal complexes identified by MLST for the 98 isolates collected at Strasbourg University Hospital. Within these CCs, the most prevalent STs were the primary founders of these complexes, ST6 (n = 14) and ST3 (n = 26) respectively. Interestingly, a particularly prevalent clone (n = 23), including virulent and carriage isolates, was identified by both MLST (ST3) and MLVA/TRST analysis (MT1/TRT1), even if the discriminatory power of these latter typing methods is high. Thus, whole genome (wg) sequencing, and particularly wgMLST, wgTRST and/or virulome determination, would be useful to identify markers to distinguish between such isolates. On the other hand, the fact that these isolates have been regularly identified from different patients and different hospital wards over a 24-month period suggests that this could be a clone capable of long-term persistence. While ST3 has already been identified^4,16^, this present study is the first to describe it as a persistent clone. This has already been described by Cheng *et al*.^35^, who identified a major endemic ST6 clone of oxacillin-resistant *S*. *lugdunensis* in a tertiary medical center in Taiwan. However, unlike Cheng *et al*., ST3 isolates are not multiresistant to antibiotics, so their persistence cannot be directly related to antibiotic pressure; it could be linked to a higher fitness under certain environmental or clinical conditions. In the light of these results and that of Kassem *et al*.^36^, who showed that clinical surfaces are frequently contaminated by *S*. *lugdunensis*, further investigations are needed to explore the mode of transmission and environmental reservoirs of this species.

In conclusion, this work provides a description of the first two VNTR-based methods for *S*. *lugdunensis* typing. MLVA and TRST are the most discriminant typing methods available to date. Furthermore, the analysis of the polymorphism of seven VNTRs in terms of variations in copy number of repeats, as well as internal sequence, allowed us to predict CCs with high accuracy while distinguishing strains belonging to some lineages. Sequence-based MLVA may prove particularly useful because it yields unambiguous data that can easily be exchanged and used for inter-laboratory comparisons. Resolving phylogenetic diversity to a high level in this clonal species, MLVA and TRST represent promising tools which could help to elucidate the global epidemiology and evolution of *S*. *lugdunensis* as well as to identify outbreaks.

## Methods

### Bacterial isolates

A total of 128 *S*. *lugdunensis* human isolates were used in this study (see Table S1 in the supplemental material). A first set of 30 isolates collected from five French regions and Sweden was used for an initial validation of two new typing methods. Among them, 20 epidemiologically unrelated isolates were used, including the reference strain ATCC 43809, 18 clinical isolates (bacteremia [n = 8], bone and joint infections [n = 4], skin and soft tissue infections [n = 3], medical device [n = 2] and other [n = 1]) as well as a carriage isolate. All 20 isolates were representative of the main clusters previously defined by MLST^16^ and MVLST^17^. To evaluate the epidemiological concordance of MLVA, we also included 5 pairs of isolates from patients at time intervals ranging from 0 to 17 days. Second, to assess the performances of MLVA, 82 clinical isolates (bone and joint infections [n = 28], skin and soft tissue infections [n = 23], bacteremia [n = 9], urinary tract infections [n = 5], medical device [n = 4] and other [n = 13]), collected during the VISLISI trial (Virulence of *Staphylococcus lugdunensis* in Severe Infections)^14^, and 16 carriage isolates (isolated from various anatomical locations not associated with infection) were included. These 98 isolates were collected from November 2013 to March 2016 at Strasbourg University Hospital, France. All the 128 isolates were identified by matrix-assisted laser desorption/ionization time-of-flight mass spectrometer.

### DNA extraction and PCR amplification

Isolates (stored at −80 °C before use) were grown overnight at 37 °C on tryptic soy agar with 5% horse blood agar plate. DNA was extracted using the InstaGene Matrix kit (Bio-Rad, Marnes la Coquette, France) according to the manufacturer’s recommendations. PCRs were performed on a Veriti Thermal Cycler (Applied Biosystems, Foster City, CA, USA) in a final volume of 25 µl containing 12.5 µl GoTaq G2 Green Master Mix (Promega, Charbonnières-Les-Bains, France), 0.50 µM each primer and 5 µl of extracted DNA.

### MultiLocus Sequence Typing (MLST)

MLST genotyping was performed by sequencing seven gene loci (*aroE*, *dat*, *ddl*, *gmk*, *ldh*, *recA* and *yqiL*) as previously described^16^, except that the PCR products were purified and sequenced by GATC Biotech SARL (Konstanz, Germany). Allelic profiles and corresponding sequence types (STs) were assigned with BioNumerics software (Version 7.6, Applied Maths, Sint-Martens-Latem, Belgium). The sequences of all new alleles and the new STs identified were entered into the international MLST database (http://bigsdb.web.pasteur.fr/staphlugdunensis/). STs were clustered into clonal complexes (CCs) when they shared at least five identical alleles (single [SLV] or double locus variants [DLV]) with another member of the group, by using the eBURST V3 program (http://eburst.mlst.net).

### MultiVirulence-Locus Sequence Typing (MVLST)

MVLST genotyping was based on the sequence determination of the intragenic regions of three virulence-associated loci, *atlLR3*, *isdJ*, and SLUG_16930 as previously described^17^, except that the PCR products were purified and sequenced by GATC Biotech SARL (Konstanz, Germany). Sequences of the three loci were analyzed using BioNumerics software (Version 7.6, Applied Maths, Sint-Martens-Latem, Belgium) to determine allelic profiles and trilocus virulence type (VT^T^).

### Multiple-Locus VNTR Analysis (MLVA) assay development

The genome sequences of *S*. *lugdunensis* isolates N920143^28^ and HKU09-01^37^ were screened *in silico* for the presence of tandem repeats (TRs) by using Tandem Repeats Finder software, version 4.07b^38^. Primers flanking 28 potential VNTR loci were designed using the OLIGO Primer Design software (Molecular Biology Insights, USA); their specificity was evaluated by Blastn. Primers were tested on the panel of 30 genetically diverse *S*. *lugdunensis* isolates to assess amplification ability, repeatability and the level of polymorphism of these loci. A VNTR was validated as a good candidate for MLVA typing if the locus (i) was present in all isolates, (ii) exhibited a minimum size of 18 bp for an individual TR, and (iii) required a single primer pair for amplification and sequencing of the internal fragment. Of the 28 VNTRs tested, seven fulfilled the above mentioned criteria, and were named SLU (for *S*. *lugdunensis*) followed by a number.

The primers used for VNTR amplification and sequencing are presented in Table 1. VNTR loci were amplified in simplex PCRs using the following steps: denaturation at 95 °C for 5 min, followed by 35 cycles of amplification including denaturation at 95 °C for 30 s, annealing at a temperature depending on the primer pair for 30 s, and extension at 72 °C for 30 s. A final extension step was performed at 72 °C for 5 min. All PCR products were visualized using gel-electrophoresis, purified, and sequenced by GATC Biotech SARL (Konstanz, Germany).

The number of TRs of each VNTR was determined with BioNumerics software (Version 7.6, Applied Maths, Saint-Martens-Latem, Belgium) by using the “polymorphic VNTR typing” plugin. To check that the entire repeat array had been sequenced, the flanking sequences (start and stop pattern) were spotted but were not included as typing information. For incomplete repeats, the TR copy number was rounded down to the nearest complete copy number, in accordance with the guidelines published by Nadon *et al*.^39^. The numbers of TRs at each locus were combined and listed in the order SLU1-SLU2-SLU3-SLU4-SLU5-SLU6-SLU7 e.g. 1-2-10-4-3-2-2. Each unique combination was converted into a distinct MLVA type (MT).

### Tandem Repeat Sequence Typing (TRST) of VNTR loci

Sequences of TR were determined for each VNTR, and each unique repeat sequence was identified by the name of the VNTR in lower case, followed by a number (Table S2); for example repeat sequence slu1_01 (Fig. S1). Each unique combination of TR sequences defined an allele (Table S3). Each allele was named by a number, corresponding to the number of TRs, followed by a lowercase letter, corresponding to the combination of the different TR sequences of the VNTR, e.g. SLU1_2a (slu1_00-slu1_01) (for ease of viewing, the allele does not include the lowercase “vntr” for each repeat; e.g. 00–01) (Fig. S1). The assignment of VNTR alleles was performed with BioNumerics software (Version 7.6, Applied Maths, Sint-Martens-Latem, Belgium), based on the successive occurrence of user-defined TR sequences. Each unique combination of alleles defined an allelic profile (with VNTRs listed in the same order as MLVA profiles), converted into a distinct TRST type (TRT).

### Data analysis

All typing data were uploaded into the BioNumerics software (Version 7.6, Applied Maths, Sint-Martens-Latem, Belgium) and clustered using the appropriate settings. Two different techniques were used to represent the relationships between isolates. A dendrogram was generated based on the categorical coefficient provided by the BioNumerics software and the algorithm of unweighted pair group method with arithmetic mean (UPGMA). Cutoff values of 67% and 91% similarity were applied to define respectively MLVA and TRST clusters. Minimum spanning trees were constructed using the categorical coefficient and the priority rule to first link types that have the highest number of single locus variants. MTs were grouped into MLVA complexes (MCs) if they differed by no more than a single VNTR. To generate a minimum spanning tree based on TRST data, a composite dataset was constructed using BioNumerics software, based on a multiple alignment of each VNTR allele.

The discriminatory ability as well as the congruence of the results obtained by using the different typing methods and/or their combination were evaluated. To this aim, both the Simpson’s diversity index (DI)^40^ and adjusted Wallace coefficients (AW)^41^ (plus confidence intervals (CI) as described by Grundmann *et al*.^42^) were calculated by resorting to the online tool at http://www.comparingpartitions.info/?link=Tool.

The standardized index of association (*I*_A_^S^) between alleles was calculated to test for linkage disequilibrium between alleles of MLVA and TRST using the LIAN Linkage Analysis 3.7 online tool (http://guanine.evolbio.mpg.de) as described by Haubold and Hudson^43^. The null hypothesis of linkage equilibrium, *I*_A_^S^ = 0, was tested with 100000 Monte Carlo simulations.

### Test of VNTR stability

Since VNTR regions are considered to be one of the fastest evolving sequences of a genome, it was important to test the stability of the selected VNTR regions. To determine this stability, two isolates were subcultured on tryptic soy agar for 30 consecutive days by streaking a single colony from each strain on agar plates. The original culture, subculture numbers 10, 20 and 30 were used for DNA extraction and the total DNA was subjected to the MLVA and TRST assays.

## Electronic supplementary material

Supplementary information
