# Supplementary material for: Multiple-Locus Variable Number Tandem Repeat Analysis (MLVA) and Tandem Repeat Sequence Typing (TRST), helpful tools for subtyping Staphylococcus lugdunensis
Source: Sci Rep. 2018 Aug 3;8:11669. doi: 10.1038/s41598-018-30144-y (PMC6076266; doi:10.1038/s41598-018-30144-y)

Supplementary Information

Multiple-Locus Variable Number Tandem Repeat Analysis (MLVA) and Tandem Repeat Sequence Typing (TRST), helpful tools for subtyping *Staphylococcus lugdunensis*

Sandrine Dahyot, Jérémie Lebeurre, Xavier Argemi, Patrice François, Ludovic Lemée, Gilles Prévost, Martine Pestel-Caron.

Supplementary Table S1. 128 *S. lugdunensis* isolates included in this study.

Isolate ID	City	Clinical sources	MLST		MVLST	MLVA			TRST	
			Sequence type (ST)	Clonal Complex (CC)	Trilocus Virulence type (VT^T)	MLVA type (MT)	MLVA complexe (MC)	MLVA cluster (67%)	TRST type (TRT)	TRST cluster (91%)
**SL_V01**	Strasbourg	Deep infection	5	5	23	7	5	IV	10	IV
**SL_V02**	Strasbourg	Osteoarticular on material	1	1	1	17	3	V	41	VII
**SL_V03**	Strasbourg	Skin and soft tissue	3	3	16	1	2	I	1	I
**SL_V04**	Strasbourg	Material device	3	3	16	1	2	I	1	I
**SL_V05**	Strasbourg	Deep infection	1	1	1	42	3	V	40	VII
**SL_V06**	Strasbourg	Deep infection	2	2	15	2	2	I	2	I
**SL_V07**	Strasbourg	Deep infection	6	1	1	12	1	II	8	V
**SL_V08**	Strasbourg	Skin and soft tissue	15	1	1	6	1	II	47	II
**SL_V09**	Strasbourg	Skin and soft tissue	6	1	1	12	1	II	8	V
**SL_V10**	Strasbourg	Material device	3	3	16	1	2	I	1	I

**SL_V11**	Strasbourg	Skin and soft tissue	5	5	23	8	5	IV	7	IV
**SL_V12**	Strasbourg	Skin and soft tissue	5	5	23	8	5	IV	7	IV
**SL_V13**	Strasbourg	Osteoarticular on material	12	1	1	4	1	II	3	II
**SL_V14**	Strasbourg	Catheter related bacteremia	24	6	13	32	4	III	22	III
**SL_V15**	Strasbourg	Osteoarticular on material	2	2	15	2	2	I	16	I
**SL_V16**	Strasbourg	Skin and soft tissue	12	1	1	44	1	II	45	II
**SL_V17**	Strasbourg	Osteoarticular	6	1	1	29	1	II	21	II
**SL_V18**	Strasbourg	Deep infection	24	6	13	3	4	III	4	III
**SL_V19**	Strasbourg	Osteoarticular on material	2	2	15	2	2	I	2	I
**SL_V20**	Strasbourg	Skin and soft tissue	24	6	34	27			30	III
**SL_V21**	Strasbourg	Catheter related bacteremia	3	3	16	1	2	I	1	I
**SL_V22**	Strasbourg	Endocarditis	24	6	13	9	4	III	24	III
**SL_V23**	Strasbourg	Osteoarticular on material	24	6	13	28	4	III	26	
**SL_V24**	Strasbourg	Osteoarticular on material	12	1	1	6	1	II	46	II
**SL_V25**	Strasbourg	Osteoarticular on material	5	5	23	20	5	IV	12	IV
**SL_V26**	Strasbourg	Skin and soft tissue	3	3	16	22	2	I	13	I
**SL_V27**	Strasbourg	Osteoarticular on material	12	1	1	35	1	II	33	II
**SL_V28**	Strasbourg	Material device	24	6	13	3	4	III	5	III
**SL_V29**	Strasbourg	Urinary	24	6	13	3	4	III	5	III
**SL_V30**	Strasbourg	Catheter related bacteremia	31	5	23	19		IV	25	
**SL_V31**	Strasbourg	Urinary	3	3	16	1	2	I	1	I

**SL_V32**	Strasbourg	Skin and soft tissue	6	1	1	4	1	II	3	II
**SL_V33**	Strasbourg	Deep infection	6	1	1	39	1	II	28	V
**SL_V34**	Strasbourg	Skin and soft tissue	4	4	29	36	6	VI	48	VI
**SL_V35**	Strasbourg	Catheter related bacteremia	1	1	1	40			42	
**SL_V36**	Strasbourg	Osteoarticular	12	1	1	4	1	II	3	II
**SL_V37**	Strasbourg	Endocarditis	1	1	1	30	3	V	18	VIII
**SL_V38**	Strasbourg	Osteoarticular on material	6	1	1	33	1	II	20	V
**SL_V39**	Strasbourg	Skin and soft tissue	24	6	13	3	4	III	5	III
**SL_V40**	Strasbourg	Osteoarticular on material	1	1	1	41	3	V	43	VII
**SL_V41**	Strasbourg	Skin and soft tissue	3	3	16	1	2	I	1	I
**SL_V42**	Strasbourg	Urinary	24	6	13	3	4	III	4	III
**SL_V43**	Strasbourg	Osteoarticular	24	6	13	3	4	III	4	III
**SL_V44**	Strasbourg	Osteoarticular	6	1	1	4	1	II	3	II
**SL_V45**	Strasbourg	Osteoarticular	24	6	13	3	4	III	4	III
**SL_V46**	Strasbourg	Deep infection	24	6	37	9	4	III	27	III
**SL_V47**	Strasbourg	Osteoarticular	3	3	16	1	2	I	1	I
**SL_V48**	Strasbourg	Deep infection	4	4	29	37	6	VI	49	VI
**SL_V49**	Strasbourg	Osteoarticular	2	2	15	2	2	I	2	I
**SL_V50**	Strasbourg	Osteoarticular on material	3	3	16	1	2	I	1	I
**SL_V51**	Strasbourg	Osteoarticular	6	1	1	10	1	II	35	II
**SL_V52**	Strasbourg	Osteoarticular on material	1	1	1	31	3	V	19	VIII
**SL_V53**	Strasbourg	Osteoarticular	2	2	15	2	2	I	2	I
**SL_V54**	Strasbourg	Osteoarticular	5	5	23	18	5	IV	23	
**SL_V55**	Strasbourg	Osteoarticular	31	5	23	7	5	IV	11	IV
**SL_V56**	Strasbourg	Material device	3	3	16	2	2	I	17	I

**SL_V57**	Strasbourg	Deep infection	1	1	1	6	1	II	44	II
**SL_V58**	Strasbourg	Catheter related bacteremia	4	4	29	14	6	VI	50	VI
**SL_V59**	Strasbourg	Osteoarticular on material	3	3	16	1	2	I	1	I
**SL_V60**	Strasbourg	Skin and soft tissue	2	2	15	2	2	I	2	I
**SL_V61**	Strasbourg	Osteoarticular on material	3	3	16	1	2	I	1	I
**SL_V62**	Strasbourg	Skin and soft tissue	15	1	1	5	1	II	36	II
**SL_V63**	Strasbourg	Deep infection	2	2	15	2	2	I	2	I
**SL_V64**	Strasbourg	Skin and soft tissue	6	1	1	34	1	II	32	II
**SL_V65**	Strasbourg	Osteoarticular	28		36	26	2	VIII	15	IX
**SL_V66**	Strasbourg	Osteoarticular on material	12	1	1	4	1	II	3	II
**SL_V67**	Strasbourg	Skin and soft tissue	3	3	16	1	2	I	1	I
**SL_V68**	Strasbourg	Deep infection	2	2	15	2	2	I	2	I
**SL_V69**	Strasbourg	Urinary	28		36	23	2	VIII	14	IX
**SL_V70**	Strasbourg	Skin and soft tissue	27	7	35	13		VII	9	
**SL_V71**	Strasbourg	Skin and soft tissue	2	2	15	2	2	I	2	I
**SL_V72**	Strasbourg	Skin and soft tissue	3	3	16	1	2	I	1	I
**SL_V73**	Strasbourg	Urinary	5	5	23	21	5	IV	38	IV
**SL_V74**	Strasbourg	Bacteremia	2	2	15	24	2	I	51	I
**SL_V75**	Strasbourg	Skin and soft tissue	2	2	15	2	2	I	2	I
**SL_V76**	Strasbourg	Deep infection	3	3	16	1	2	I	1	I
**SL_V77**	Strasbourg	Catheter related bacteremia	26	7	38	38			39	
**SL_V78**	Strasbourg	Skin and soft tissue	3	3	16	1	2	I	1	I
**SL_V79**	Strasbourg	Deep infection	6	1	1	11	1	II	31	V
**SL_V80**	Strasbourg	Osteoarticular on material	6	1	1	5	1	II	6	II

**SL_V81**	Strasbourg	Skin and soft tissue	6	1	1	5	1	II	6	II
**SL_V82**	Strasbourg	Skin and soft tissue	1	1	1	10	1	II	34	II
**SL_C01**	Strasbourg	Carriage	3	3	16	1	2	I	1	I
**SL_C02**	Strasbourg	Carriage	6	1	1	11	1	II	29	V
**SL_C03**	Strasbourg	Carriage	3	3	16	1	2	I	1	I
**SL_C04**	Strasbourg	Carriage	3	3	16	1	2	I	1	I
**SL_C05**	Strasbourg	Carriage	3	3	14	1	2	I	1	I
**SL_C06**	Strasbourg	Carriage	3	3	16	25	2	I	37	I
**SL_C07**	Strasbourg	Carriage	26	7	38	43	2	VIII	52	
**SL_C08**	Strasbourg	Carriage	3	3	16	1	2	I	1	I
**SL_C09**	Strasbourg	Carriage	12	1	1	4	1	II	3	II
**SL_C10**	Strasbourg	Carriage	6	1	1	5	1	II	6	II
**SL_C11**	Strasbourg	Carriage	2	2	15	2	2	I	2	I
**SL_C12**	Strasbourg	Carriage	27	7	35	13		VII	9	
**SL_C13**	Strasbourg	Carriage	3	3	16	1	2	I	1	I
**SL_C14**	Strasbourg	Carriage	3	3	16	1	2	I	1	I
**SL_C15**	Strasbourg	Carriage	3	3	16	1	2	I	1	I
**SL_C16**	Strasbourg	Carriage	3	3	16	1	2	I	1	I
**SL_T01**	Rouen	Osteoarticular	13		27	49			60	
**SL_T02**	Kronoberg	Carriage	1	1	1	52	3	V	61	VIII
**SL_T03**	Rouen	Endocarditis	8	2	9	2	2	I	58	I
**SL_T04**	Rouen	Deep infection	10	6	13	53	4	III	62	III
**SL_T05**	Rouen	Osteoarticular on material	4	4	28	14	6	VI	67	VI
**SL_T06**	Rouen	Bacteremia	9	4	11	55		VII	68	
**SL_T07**	Rouen	Catheter related bacteremia	16	3	16	50			59	I
**SL_T08**	Nantes	Material device	3	3	16	1	2	I	1	I
**SL_T09**	Nantes	Osteoarticular	14	2	15	2	2	I	2	I

**SL_T10**	Nantes	Skin and soft tissue	6	1	2	15	1	II	64	II
**SL_T11**	Bordeaux	Bacteremia	17	4	29	51	6	VI	66	VI
**SL_T12**	Bordeaux	Bacteremia	18	5	24	7	5	IV	10	IV
**SL_T13**	Bordeaux	Bacteremia	7	1	4	54			65	
**SL_T14**	Nancy	Bacteremia	19	2	17	47	2	I	56	I
**SL_T15**	Nancy	Skin and soft tissue	12	1	5	4	1	II	3	II
**SL_T16**	Nancy	Osteoarticular	20	3	16	48	2	I	57	I
**SL_T17**	Montpellier	Deep infection	15	1	2	15	1	II	69	II
**SL_T18**	Montpellier	Osteoarticular on material	2	2	17	2	2	I	2	I
**SL_T19**	Rouen	Bacteremia	5	5	23	46			53	
**SL_AP1**	Rouen	Osteoarticular	3	3	16	1	2	I	1	I
**SL_AP2**	Rouen	Osteoarticular	3	3	16	1	2	I	1	I
**SL_CB1**	Rouen	Bacteremia	15	1	1	16	1	II	55	II
**SL_CB2**	Rouen	Bacteremia	15	1	1	16	1	II	55	II
**SL_CJ1**	Rouen	Deep infection	24	6	13	3	4	III	63	III
**SL_CJ2**	Rouen	Bacteremia	24	6	13	3	4	III	63	III
**SL_DJ1**	Rouen	Endocarditis	32	2	15	2	2	I	2	I
**SL_DJ2**	Rouen	Endocarditis	32	2	15	2	2	I	2	I
**SL_DSM**		Skin and soft tissue	24	6	13	45	2	I	54	
**SL_LJ1**	Rouen	Deep infection	2	2	15	2	2	I	2	I
**SL_LJ2**	Rouen	Deep infection	2	2	15	2	2	I	2	I

Supplementary Table S2. Individual TR sequences identified from 128 *S. lugdunensis* isolates used in the study.

SLU1	TR sequences
>slu1_01	GGGGCCCCAACAAAGAGAAATGCGAAAAGCATTTCACCAAGCAAAGCAAGTTGGGGT
>slu1_02	GGGGCCCCAACAAAGAGAAATGCGAAAAGCATTTCGCCAAGCAAAGCAAGTTGGGGT
>slu1_03	GGGGCCCCAACAAAGAGAAATGCGAAAAACATTTCACCAAGCAAAGCAAGTTGGGGT

SLU2	TR sequences
>slu2_00	GGGGCCCCAGCACAGAGAAATGCAACAAACATTTCTACGTGCAACGCAAGCTGGGGTG
>slu2_01	GGGCCCTCAGCACAGAGAAATGCACCAAGCATTTCTACGGACAACGCAAGCTGGGGTG
>slu2_02	GGGCCCTCAGCACAAAGAAATGCACCAAGCATTTCTACGGACAATGCAAGCTGGGGTG
>slu2_03	GGGGCCCCAGCACAAAGAAATGCACCAAGCATTTCTACGTGCAACGCAAGCTGGGGGT
>slu2_04	GGGGCCCCAGCACAAAGAAATGCACCAAGCATTTCTACGTGCAACGCAAGCTGGGGTG
>slu2_05	GGGGCCTCAGCACAGAGAAATGCAACAAACATTTCTACGTGCAACGCAAGCTGGGGTG
>slu2_06	GGGGCCTCAGCACAGAGAAATGCACCAAGCATTTCTACGGACAACGCAAGCTGGGGTG
>slu2_07	GGGCCCTAAGCACAGAGAAATGCACCAAGCATTTCTACGGACAACGCAAGCTGGGGTG
>slu2_08	GGGGCCCCAGCACAGAGAAATGCACCAAGCATTTCTACGGACAACGCAAGCTGGGGTG
>slu2_09	GGGGCCCCAGCACAAAGAAATGCGACAAACATTTCTACGGACAACGCAAGCTGGGGTG
>slu2_10	GGGCCCTCAGCACAGAGAAATGCAACAAACATTTCTACAGACAACGCAAGCTGGGGGTG
>slu2_11	GGGGCCCCAGCACGTAGAAATGCACCAAGCATTTCTACGTGCAACGCAAGCTGGGGTG
>slu2_12	GGGGCCCCAGCACAAAGAAATGCACCAAGCATTTCTACGGACAACGCAAGCTGGGGGT
>slu2_13	GGGGCCCCAGCACAGAGAAATGCACCAAGCATTTCTACGGACAACGCAAGCTGGGGGTGG
>slu2_14	GGGGCCCCAGCACAGAGAAATGCAACAAACATTTCTACGTGCAACGCAAGCTGGGGT

SLU3	TR sequences
>slu3_00	ACAATGAAGGCAAAGACAACAGACCAAATAAGCCATCGGATGGTACGG
>slu3_01	ACAATGAAGGCAAAGACAACAAGCCAAACAAGCCATCAGATGGTACAG
>slu3_02	ATAACGGAGGCAAAGATAACAAGCCAAACAAACCATCAGATGGCACGG
>slu3_03	ATAACGGAGGCAAAGATAACAGGCCAAACAAACCATCAGATGGCACGG
>slu3_04	ATAACGGAGGCAAAGATAACAGGCCAAACAAACCATCGGATGGTACGG
>slu3_05	ATAACGGAGGCAAAGATAACAGGCCAAACAAACCATCGAATGGTACGG
>slu3_06	ATAACGGAGGCAAAGATAACAGGCCAAACAAACCATCAGATGGCATGG
>slu3_07	ATAACGTAGGCAAAGATAACAAGCCAAACAAACCATCAGATGGCACGG
>slu3_08	ACAATGAAGGCAAAGATAACAAGCCAAACAAACCATCGGATGGCACGG
>slu3_09	ACCATGGAGGTAAAGATAACAAGCCAAACAAACCATCAGATGGCACGG
>slu3_10	ACCATGGAGGCAAAGACAACAGACCAAATAAGCCATCGGATGGTACGG
>slu3_11	ACCACGGAGGCAAAGATAACAAGCCAAACAAACCATCGGATGGCACAG
>slu3_12	ACCACGGAGGCAAAGATAACAAGCCAAACAAACCATCAGATGGTACGG
>slu3_13	ACCATGGAGGTAAAGACAACAAGCCA
>slu3_14	ATAACGGAGGCAAAGACAACAAGCCAAACAAGCCATCGGATGGTACGG
>slu3_15	ATAACGGAGGCAAAGATAACAAGCCAAACAAACCATCAGATGGTACGG
>slu3_16	ATAATGGAGGCAAAGATAACAAACCAAACAAGCCATCGGATGGCACGG
>slu3_17	ATAACGGAGGCAAAGATAACAGGCCAAACAAACCATCGGATGGTACGA

>slu3_18	ATAACGGAGGCAAAGATAACAAGCCAAACAAGCCATCGGATGGTACGG
>slu3_19	ATAACGGAGGCAAAGATAACAAGCCAAACAAACCATCGGATGGTACGG
>slu3_20	ATAACGGAGGTAAAGATAACAAGCCA
>slu3_21	ACCATGGAGGTAAAGATAACAAGCCAAACAAGCCATCAGATGGCACGG
>slu3_22	ATCATGGAGGTAAAGACAACAAGCCA
>slu3_23	ACAATGAAGGCAAAGACAACAAGCCAAACAAGCCATCAGATGGTACGG
>slu3_24	ATAATGGAGGCAAAGATAACAAACCAAACAAGCCATCGGATGGTACGG
>slu3_25	ACAATGAAGGCAAAGACAACAAGCCAAACAAGCCATCGGATGGTACGG
>slu3_26	ATAACGGAGGCAAAGATAACAAACCAAACAAACCATCAGATGGTACGG
>slu3_27	ATAACGGAGGCAAAGATAACAAGCCAAACAAACCATCTGATGGCACGG
>slu3_28	ACAATGAAGGCAAAGATAACAGGCCAAACAAACCATCAGATGGCACGG
>slu3_29	ACAATGAAGGCAAAGATAACAAGCCAAACAAGCCATCGGATGGTACGG
>slu3_30	ATAACGGAGGCAAAGATAACAAGCCAAACAAACCATCGGATGGCACGG
>slu3_31	ATAACGGAGGCAAAGACAACAAGCCAAACAAACCATCGGATGGCACAG
>slu3_32	ACCATGGAGGCAAAGACAACAAGCCAAACAAGCCATCAGATGGTACAG
>slu3_33	ATAACGGAGGCAAAGATAACAAACCAAACAAGCCATCGGATGGCACGG
>slu3_34	ATAACGGAGGCAAAGACAACAAGCCAAACAAGCCATCAGATGGTACAG

SLU4	TR sequences
>slu4_00	CACCCCAGCTTGCGTTGTACGTAGAAATGCTTTTCGCATTCCTCTTTGCTGGGGCCCC
>slu4_01	CACCCCAGCTTGCGTTGTACGTAGAAGTGCTTATCGTATTCCTCGTTGCTTGGCCCT
>slu4_02	CACCCCAGCTTGCGTTGTACGTAGAAGTGCTTATCGTATTCCTCTTTGCTTGGCCCT
>slu4_03	CACCCCAGCTTGCGTTGTACGTAGAAATGCTTTTCGCATTCCTCTTTGCTTGGCCCT
>slu4_04	CATCCCAGCTTGCTTTGT
>slu4_05	CACCCCAGCTTGCTTTGT
>slu4_06	CACCCCAGCTTGCGTTGT
>slu4_07	CACCCCAGCTTGCGTTGTACGTAGAAATGCTTTTCGTATTCCTCTTTGCTTGGCCCT

SLU5	TR sequences
>slu5_00	TTCACCAAGCAAAGCAAGTTGGAGTGGGGCCCCAACACAGAGAATTTCGAAAAGAAA
>slu5_01	TTCACCAAGCAAAGCAAGTTGGGGTGGGGCCCTAACACAGAGAATTTCGAAAAGAAA
>slu5_02	TTCACCAAGCAAAGCAAGTTGGGGTGGGGCCCCAACACAGAGGCTTTCGAAGCGAAA
>slu5_03	TTCACCAAGCAAAGCAAGTTGGGGTGGGGCCCCAACACAGAGGCTTTCGAA
>slu5_04	GGCAACGGACAACGCAAGTTGGGGTGAGGCCCAA
>slu5_05	TTCACCAAGCAAAGCAAGTTGGGGTGAGGCCCAA
>slu5_06	TTCACCAAGCAAAGCAAGTTGGGGTGGGGCCCCAACAAAGAGGCTTTCGAAGCGAAA
>slu5_07	TTCACCAAGCAAAGCAAGTTGGGGTGGGGCCCCAACACAGAGAATTTCGAAAAGAAA
>slu5_08	GGCAACGGACAACGCAAGTTGGGGTGGGGCCCAA
>slu5_09	GGCAACGGACAACGCAAGTTGGGGTGAGGCTCAA
>slu5_10	TTCACCAAGCAAAGCAAGTTGGAGTGGGGCCCCAACACAGAGGTTTTCGAAGCGAAA
>slu5_11	TTCACCAAGCAAAGCAAGTTGGAGTGGGGCCCCAACACAGAGGCTTTCGAAGCGAAA
>slu5_12	TTCACCAAGCAAAGCAAGTTGGATTGGGGCCCCAACACAGAGAATTTCGAAAAGAAA
>slu5_13	TTCACCAAGCAAAGCAAGTTGGATTGGGGCCCCAACACAGAGGCTTTCGAAGCGAAA
>slu5_14	GGCAACGGACAACGCAAGTTGGGGTGGGGCCCCAACACAGAGGCTTTCGAAGCGAAA

SLU6	TR sequences
>slu6_00	CCCACCCCAACTTGCATTGTTTGTTGAAATGCTTGGCGCATTCCTTTTTGTTGGGGCC
>slu6_01	CCCACCCCAACTTGCATTGTTTGTTGAAGTGCTTGGCGCATTCCTTTTTGTTGGGGCC
>slu6_02	CCCACCCCAACTTGCGTTGTTTGTTGAAGTGCTTGGCGCATTCCTTTTTGTTGGGGCC
>slu6_03	CCCACCCCAACTTGCGTTGTTCGTTGAAATGCTTGGCGCATTCCTTTTTGTTGGGGCCCC
>slu6_04	CCCACCCCAACTTGCTGTGCTTGACGAAATGCGCTTCGCATTTCTCTCTGTTGGGGCC
>slu6_05	CCACCCCAACTTGCGTTGTTCGTTGAAATGCTTGGCGCATTCCTTTTTGTTGGGGCC
>slu6_06	CCCACCCCAACTTGCGTTGTTTGTTGAAGTGCTTGGCGCATTCCTTTTTGTTGGGGCCCC
>slu6_07	CCACCCCAACTTGCGTTGTTCGTTGAAATGCTTGGCGCATTCCTTTTTGTTGGGGCCCC
>slu6_08	CCCACCCCAACTTGCATTGTTTGTTGAAGTGCTTGGCGCATTCCTTTTTGTTGGGGCCCC
>slu6_09	CCACCCCAACTTGCGTTGTTCGTTGAAATGCTTGACGCATTCCTTTTTGTTGGGGCCCC
>slu6_10	CCCACCCCAACTTGCGTTGTTTGTTGAAGTGCTTGGCGCATTCCTTTTTGTTGAGGCC
>slu6_11	CCCACCCCAACTTGCGTTGTTTGTTGAAATGCTTGGCGCATTCCTTTTTGTTGGGGCCCC
>slu6_12	ACCCCAACTTGCGTTGTTCGTTGGCTTTCTTTTGGAAAGCCTTTTTGTTGGGGGC
>slu6_13	GCCCCACCCCAACTTGCGTTGTTCGTTGAAATGCTTGGCGCATTCCTTTTTGTTGGGGCCCC

SLU7	TR sequences
>slu7_00	TATTTAAAAATTGGTGGTTCTCTA
>slu7_01	TATTTAAAAAT

Supplementary Figure S1. Alignment of the three TRs sequences and alleles for SLU1 among study isolates.

<u>Existing TR sequences for SLU1 (57 bp)</u>:

```
                 10        20        30        40        50        60
slu1_00  GGGGCCCCAACAAAGAGAAATGCGAAAAGCATTTCACCAAGCAAAGCAAGTTGGGGT
slu1_01  ...................................G.....................
slu1_02  ............................A............................
```

<u>List of alleles for SLU1</u>:

Allele name	Number of TRs	Combination of TR sequences
SLU1_1a	1	slu1_01
SLU1_1b		slu1_00
SLU1_1c		slu1_02
SLU1_2a	2	slu1_00-slu1_01
SLU1_2b		slu1_00-slu1_00
SLU1_3a	3	slu1_00-slu1_00-slu1_01

Supplementary Table S3. List of TRST alleles identified from 128 *S. lugdunensis* isolates used in the study.

SLU1	Alleles
SLU1_1a	1
SLU1_1b	0
SLU1_1c	2
SLU1_2a	0-1
SLU1_2b	0-0
SLU1_3a	0-0-1

SLU2	Alleles
SLU2_1a	4
SLU2_1b	8
SLU2_2a	0-1
SLU2_2b	0-6
SLU2_2c	4-1
SLU2_2d	3-9
SLU2_2e	11-1
SLU2_2f	12-13
SLU2_2g	4-7
SLU2_2h	14-0
SLU2_3a	0-1-1
SLU2_3b	0-6-1
SLU2_3c	0-10-2
SLU2_3d	11-1-1
SLU2_3e	14-0-1
SLU2_4a	0-1-1-1
SLU2_4b	0-6-1-1
SLU2_4c	0-5-6-1

SLU3	Alleles
SLU3_3a	0-4-20
SLU3_4a	0-7-9-13
SLU3_4b	0-18-18-20
SLU3_4c	0-15-9-13
SLU3_5a	0-6-1-2-13
SLU3_7a	0-6-1-2-12-9-13
SLU3_8a	0-3-1-2-8-12-9-13
SLU3_8b	0-6-1-2-8-12-9-13
SLU3_8c	0-4-1-15-14-16-21-22
SLU3_9a	25-3-1-2-11-10-26-9-13
SLU3_9b	25-3-1-27-11-10-26-9-13
SLU3_9c	28-29-30-15-31-32-33-21-22

SLU3_9d	0-4-1-34-30-11-10-33-13
SLU3_9e	1-2-8-11-10-2-9-9-13
SLU3_10a	0-4-1-4-1-15-14-16-21-22
SLU3_10b	0-4-1-4-23-14-24-16-21-22
SLU3_10c	0-5-1-2-8-11-10-7-9-13
SLU3_10d	0-4-1-2-8-11-10-7-9-13
SLU3_11a	0-17-18-19-18-19-18-18-18-18-20
SLU3_11b	0-6-1-2-8-12-9-8-12-9-13
SLU3_11c	0-4-1-2-8-11-10-7-9-9-13
SLU3_11d	0-6-1-2-8-12-9-8-12-21-13

SLU4	Alleles
SLU4_1a	6
SLU4_2a	0-5
SLU4_2b	3-4
SLU4_3a	0-2-4
SLU4_3b	0-1-4
SLU4_3c	0-7-4
SLU4_4a	0-0-1-4
SLU4_4b	0-0-2-4
SLU4_5a	0-0-0-1-4
SLU4_5b	0-0-0-2-4
SLU4_6a	0-0-0-0-2-4

SLU5	Alleles
SLU5_2a	0-3
SLU5_2b	0-5
SLU5_2c	2-4
SLU5_2d	10-4
SLU5_2e	11-4
SLU5_2f	6-8
SLU5_2g	13-4
SLU5_3a	1-2-4
SLU5_3b	0-2-4
SLU5_3c	0-6-4
SLU5_3d	0-6-8
SLU5_3e	0-1-5
SLU5_3f	12-11-4
SLU5_4a	0-1-1-5
SLU5_4b	0-1-2-4
SLU5_4c	0-7-2-4
SLU5_4d	0-1-2-9
SLU5_5a	13-14-14-14-4

SLU6	Alleles
SLU6_1a	13
SLU6_2a	4-11
SLU6_2b	4-3
SLU6_2c	4-7
SLU6_2d	0-8
SLU6_2e	4-9
SLU6_3a	4-0-11
SLU6_3b	4-5-6
SLU6_3c	4-5-7
SLU6_3d	0-1-8
SLU6_3e	4-2-3
SLU6_3f	4-12-11
SLU6_4a	4-2-2-3
SLU6_5a	4-2-2-10-3

SLU7	Alleles
SLU7_2a	0-1
SLU7_3a	0-0-1
SLU7_4a	0-0-0-1

Supplementary Table S4. MLVA types and TRST types of the 128 *S. lugdunensis* isolates used in the study

<u>MLVA types</u>

MLVA type (MT)	MLVA complexe (MC)	SLU1	SLU2	SLU3	SLU4	SLU5	SLU6	SLU7
**1**	**2**	1	2	10	4	3	2	2
**2**	**2**	1	2	10	4	3	3	2
**3**	**4**	2	2	10	2	3	4	2
**4**	**1**	2	3	8	3	4	2	2
**5**	**1**	2	3	8	3	3	2	2
**6**	**1**	2	4	8	3	4	2	2
**7**	**5**	1	1	11	3	3	2	2
**8**	**5**	1	1	11	3	4	2	2
**9**	**4**	2	2	10	2	3	2	2
**10**	**1**	2	3	8	3	2	2	2
**11**	**1**	2	3	11	3	3	2	2
**12**	**1**	2	3	11	3	4	2	2
**13**		3	2	9	2	3	3	2
**14**	**6**	2	3	9	1	3	3	2
**15**	**1**	2	2	8	3	4	2	2
**16**	**1**	1	3	8	3	4	2	2
**17**	**3**	2	4	8	4	4	3	3
**18**	**5**	1	1	3	3	3	2	2
**19**		1	1	4	6	3	2	2
**20**	**5**	1	1	11	4	3	2	2
**21**	**5**	1	1	11	5	3	2	2
**22**	**2**	1	2	8	4	3	2	2
**23**	**2**	1	2	9	4	2	3	2
**24**	**2**	1	2	10	4	2	3	2
**25**	**2**	1	2	10	4	3	1	2
**26**	**2**	1	3	9	4	2	3	2
**27**		2	1	11	2	3	4	2
**28**	**4**	2	2	4	2	3	5	2
**29**	**1**	2	2	8	3	2	2	2
**30**	**3**	2	2	8	3	3	3	3
**31**	**3**	2	2	8	4	3	3	3
**32**	**4**	2	2	10	2	3	3	2
**33**	**1**	2	2	11	3	3	2	2
**34**	**1**	2	3	5	3	4	2	2
**35**	**1**	2	3	7	3	4	2	2
**36**	**6**	2	3	9	1	4	2	2
**37**	**6**	2	3	9	1	4	3	2

**38**		2	3	9	4	5	3	2
**39**	**1**	2	3	11	3	2	2	2
**40**		2	4	4	5	4	2	2
**41**	**3**	2	4	8	3	4	3	3
**42**	**3**	2	4	8	4	2	3	3
**43**	**2**	3	3	9	4	2	3	2
**44**	**1**	2	4	8	3	2	2	2
**45**	**2**	1	2	10	2	2	3	2
**46**		1	1	9	5	2	2	2
**47**	**2**	1	1	10	4	3	3	2
**48**	**2**	1	2	10	3	3	2	2
**49**		1	3	9	3	3	3	2
**50**		1	3	10	4	2	2	2
**51**	**6**	2	1	9	1	3	3	2
**52**	**3**	2	2	8	4	4	3	3
**53**	**4**	2	2	10	2	3	5	2
**54**		2	4	7	3	3	1	2
**55**		3	1	9	2	3	2	2

TRST types

TRST type (TRT)	SLU1	SLU2	SLU3	SLU4	SLU5	SLU6	SLU7
**1**	1b	2d	10a	4b	3c	2c	2a
**2**	1b	2d	10b	4b	3d	3b	2a
**3**	2a	3b	8b	3b	4b	2d	2a
**4**	2a	2c	10c	2a	3b	4a	2a
**5**	2a	2c	10d	2a	3b	4a	2a
**6**	2a	3b	8b	3b	3b	2d	2a
**7**	1a	1b	11a	3a	4c	2e	2a
**8**	2a	3b	11b	3b	4b	2d	2a
**9**	3a	2f	9d	2b	3f	3a	2a
**10**	1a	1b	11a	3a	3b	2e	2a
**11**	1a	1b	11a	3c	3b	2e	2a
**12**	1a	1b	11a	4b	3b	2e	2a
**13**	1b	2d	8c	4b	3c	2c	2a
**14**	1b	2h	9c	4a	2e	3b	2a
**15**	1b	3e	9c	4a	2e	3b	2a
**16**	1c	2d	10b	4b	3d	3b	2a
**17**	1b	2d	10a	4b	3c	3c	2a
**18**	2a	2a	8a	3b	3e	3d	3a
**19**	2a	2a	8a	4a	3e	3d	3a
**20**	2a	2b	11b	3b	3e	2d	2a
**21**	2a	2b	8b	3b	2c	2d	2a
**22**	2a	2c	10c	2a	3b	3e	2a
**23**	1a	1b	3a	3a	3b	2e	2a
**24**	2a	2c	10d	2a	3b	2b	2a
**25**	1a	1b	4b	6a	3b	2e	2a
**26**	2a	2c	4a	2a	3b	5a	2a
**27**	2a	2e	10c	2a	3b	2b	2a
**28**	2a	3b	11b	3b	2c	2d	2a
**29**	2a	3b	11b	3b	3e	2d	2a
**30**	2a	1a	11c	2a	3b	4a	2a
**31**	2a	3b	11d	3b	3e	2d	2a
**32**	2a	3b	5a	3b	4b	2d	2a
**33**	2a	3b	7a	3b	4b	2d	2a
**34**	2a	3b	8a	3b	2c	2d	2a
**35**	2a	3b	8b	3b	2d	2d	2a
**36**	2a	3b	8b	3b	3a	2d	2a
**37**	1b	2d	10a	4b	3c	1a	2a
**38**	1a	1b	11a	5b	3b	2e	2a
**39**	2a	3d	9e	4a	5a	3e	2a
**40**	2a	4a	8a	4a	2b	3d	3a

**41**	2a	4a	8a	4a	4a	3d	3a
**42**	2a	4b	4c	5a	4d	2d	2a
**43**	2a	4b	8a	3b	4a	3d	3a
**44**	2a	4b	8a	3b	4b	2d	2a
**45**	2a	4b	8b	3b	2c	2d	2a
**46**	2a	4b	8b	3b	4b	2d	2a
**47**	2a	4c	8b	3b	4b	2d	2a
**48**	2b	3c	9a	1a	4c	2x	2a
**49**	2b	3c	9a	1a	4c	3f	2a
**50**	2b	3c	9b	1a	3b	3f	2a
**51**	1b	2d	10b	4b	2f	3b	2a
**52**	3a	3d	9e	4a	2g	3e	2a
**53**	1a	1b	9g	5c	2e	2e	2c
**54**	1a	2c	10d	2a	2c	3e	2a
**55**	1a	3b	8b	3b	4b	2d	2a
**56**	1b	1c	10b	4b	3d	3b	2a
**57**	1b	2d	10a	3a	3c	2c	2a
**58**	1b	2d	10e	4b	3d	3b	2a
**59**	1b	3b	10a	4b	2h	2c	2a
**60**	1b	3f	9f	3d	3g	3g	2b
**61**	2a	2a	8a	4a	4a	3d	3a
**62**	2a	2c	10d	2a	3b	5b	2a
**63**	2a	2g	10d	2a	3b	4a	2a
**64**	2a	2i	8b	3b	4b	2d	2a
**65**	2a	4b	7b	3b	3b	1b	2a
**66**	2b	1c	9b	1a	3b	3f	2a
**67**	2b	3c	9a	1a	3b	3f	2a
**68**	3a	1d	9h	2c	3b	2f	2a
**69**	2a	2d	8b	3b	4b	2d	2a

Supplementary Figure S2. TRST clustering of the 128 isolates of *S. lugdunensis* by the UPGMA method. The names of isolates, MLST sequence type (ST), MLST clonal complex (CC), MVLST trilocus virulence type (VT^T), MLVA type (MT), TRST type (TRT), the clinical sources and the city of strains are shown on the right. A cutoff value of 91% similarity was applied to define TRST clusters (named TRST cluster I to IX). The colors used are based on MLVA complexes (MCs).

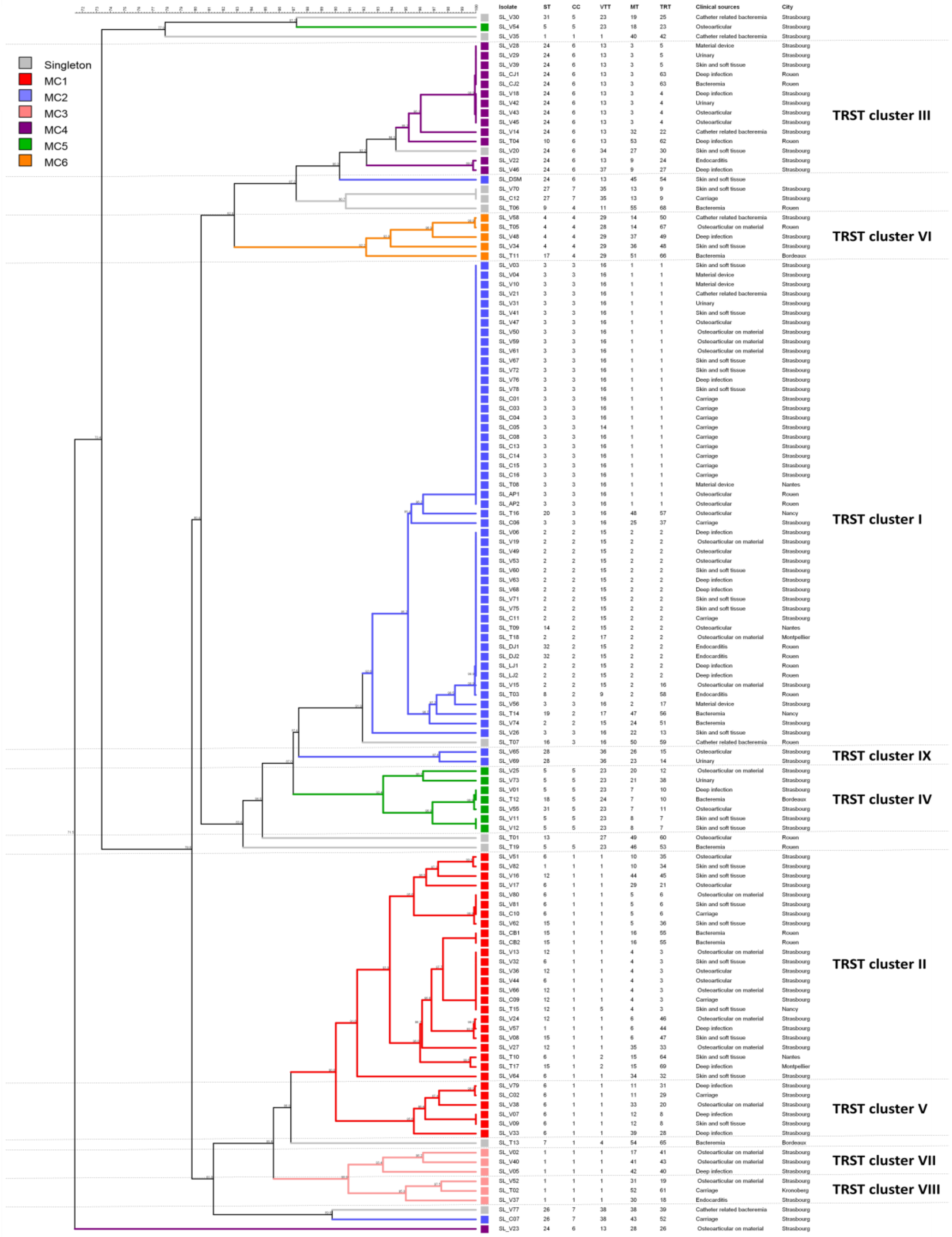

Supplement: Supplementary file 1 — Supplementary information [file 41598_2018_30144_MOESM1_ESM.pdf]
